# Physiological changes during torpor favor association with *Endozoicomonas* endosymbionts in the urochordate *Botrylloides leachii*

**DOI:** 10.3389/fmicb.2023.1072053

**Published:** 2023-05-31

**Authors:** Yosef Hyams, Maxim Rubin-Blum, Amalia Rosner, Leonid Brodsky, Yuval Rinkevich, Baruch Rinkevich

**Affiliations:** ^1^Israel Oceanographic and Limnological Research, National Institute of Oceanography, Haifa, Israel; ^2^Department of Marine Biology, Leon H. Charney School of Marine Sciences, University of Haifa, Haifa, Israel; ^3^Tauber Bioinformatics Research Center, University of Haifa, Haifa, Israel; ^4^Sagol Department of Neurobiology, University of Haifa, Haifa, Israel; ^5^Comprehensive Pneumology Center, Institute of Lung Biology and Disease, Helmholtz Zentrum München, Munich, Germany

**Keywords:** ascidians, torpor, hibernation, aestivation, symbiosis, *Endozoicomonas*, metabolism

## Abstract

Environmental perturbations evoke down-regulation of metabolism in some multicellular organisms, leading to dormancy, or torpor. Colonies of the urochordate *Botrylloides leachii* enter torpor in response to changes in seawater temperature and may survive for months as small vasculature remnants that lack feeding and reproductive organs but possess torpor-specific microbiota. Upon returning to milder conditions, the colonies rapidly restore their original morphology, cytology and functionality while harboring re-occurring microbiota, a phenomenon that has not been described in detail to date. Here we investigated the stability of *B. leachii* microbiome and its functionality in active and dormant colonies, using microscopy, qPCR, *in situ* hybridization, genomics and transcriptomics. A novel lineage of *Endozoicomonas*, proposed here as *Candidatus* Endozoicomonas endoleachii, was dominant in torpor animals (53–79% read abundance), and potentially occupied specific hemocytes found only in torpid animals. Functional analysis of the metagenome-assembled genome and genome-targeted transcriptomics revealed that *Endozoicomonas* can use various cellular substrates, like amino acids and sugars, potentially producing biotin and thiamine, but also expressing various features involved in autocatalytic symbiosis. Our study suggests that the microbiome can be linked to the metabolic and physiological states of the host, *B. leachii*, introducing a model organism for the study of symbioses during drastic physiological changes, such as torpor.

## 1. Introduction

Dormancy (i.e., torpor) is widespread and well-documented across terrestrial and marine animals (Hand and Hardewig, 1996; Cáceres, 1997; Storey, 2002; Storey and Storey, 2011). Metazoans undergo dramatic changes during torpor, including metabolic depression, arrested development, and phenotypic plasticity. This reduction in host metabolism, body temperature and activity may affect the holobiont, altering the ecological landscape and selection of microbial community that is associated with the host. Whereas these associated microbes are considered crucial for the functionality and health of the metazoans (Berg et al., 2020), we know little about their diversity and function during the transition between active and dormant stages in torpor hosts. Studies in mammalian winter torpor cases, such as in the Syrian hamster, the ground squirrel and the brown bear, revealed restructuring of gut microbiota (Sonoyama et al., 2009; Carey et al., 2013; Sommer et al., 2016). Amphibian dormancy is associated with a decline in bacterial counts, changes in gut microbiota composition and the contribution of bacteria to energy balance (Carr et al., 1976; Gossling et al., 1982; Wiebler et al., 2018). Yet, most torpor studies focus on gut microbes in vertebrates, whereas how these transitions affect other tissues and basal metazoan lineages are poorly understood (Carey et al., 2013; Kohl et al., 2014; Lindsay et al., 2020; Sze et al., 2020).

Ascidians, or sea squirts (Tunicata), are a group of filter-feeding marine invertebrates that hold an important evolutionary position as a sister clade to the chordates (Vanni et al., 2022). They present a wide range of torpor events in various colonial taxa (Mukai et al., 1983; Turon, 1992; De Caralt et al., 2002; Hyams et al., 2017). As in many other sedentary marine organisms, symbiotic bacteria are known to reside in tunicates and are also known as tissue-associated, such as in endodermal tissues, the pharynx, the tunic and the intestine (Behrendt et al., 2012; Dishaw et al., 2014). The ascidian microbiota contributes to nutrition via photosynthesis (Donia et al., 2011; López-Legentil et al., 2011; Hirose, 2015), production of secondary metabolites (Schmidt, 2014; Tianero et al., 2015; Chen et al., 2018), nitrification (Martínez-García et al., 2008; Erwin et al., 2014), protection against biofouling, predation (Degnan et al., 1989; Paul et al., 1990; Fu et al., 1998; Vervoort et al., 1998; Faulkner, 2001; Schreiber et al., 2016), vanadium accumulation (Ueki et al., 2019), and the host immune response (Liu et al., 2021).

One key bacterial symbiont of ascidians is *Endozoicomonas* gammaproteobacterium (order Pseudomonadales, family Endozoicomonadaceae; Schreiber et al., 2016), a clade of intracellular or cell-associated facultative bacteria, found in close association with diverse marine invertebrate hosts, including reef-building corals, sponges, bryozoans, sea squirts, sea slugs, and mollusks (Jensen et al., 2010; Forget and Juniper, 2013; Morrow et al., 2015; Bourne et al., 2016; Miller et al., 2016; Neave et al., 2016; Schreiber et al., 2016; Pogoreutz et al., 2022). Despite the abundance and worldwide distribution of *Endozoicomonas* in multiple marine organisms, our knowledge of their functions and symbiotic relationships is limited. Here we aimed to study the bacterial diversity associated with the torpor state of the colonial tunicate *Botrylloides leachii*, representing one of the most dramatic torpor phenomena in the Chordata (Hyams et al., 2017), focusing on the key *Endozoicomonas* symbionts.

**Figure 1:**
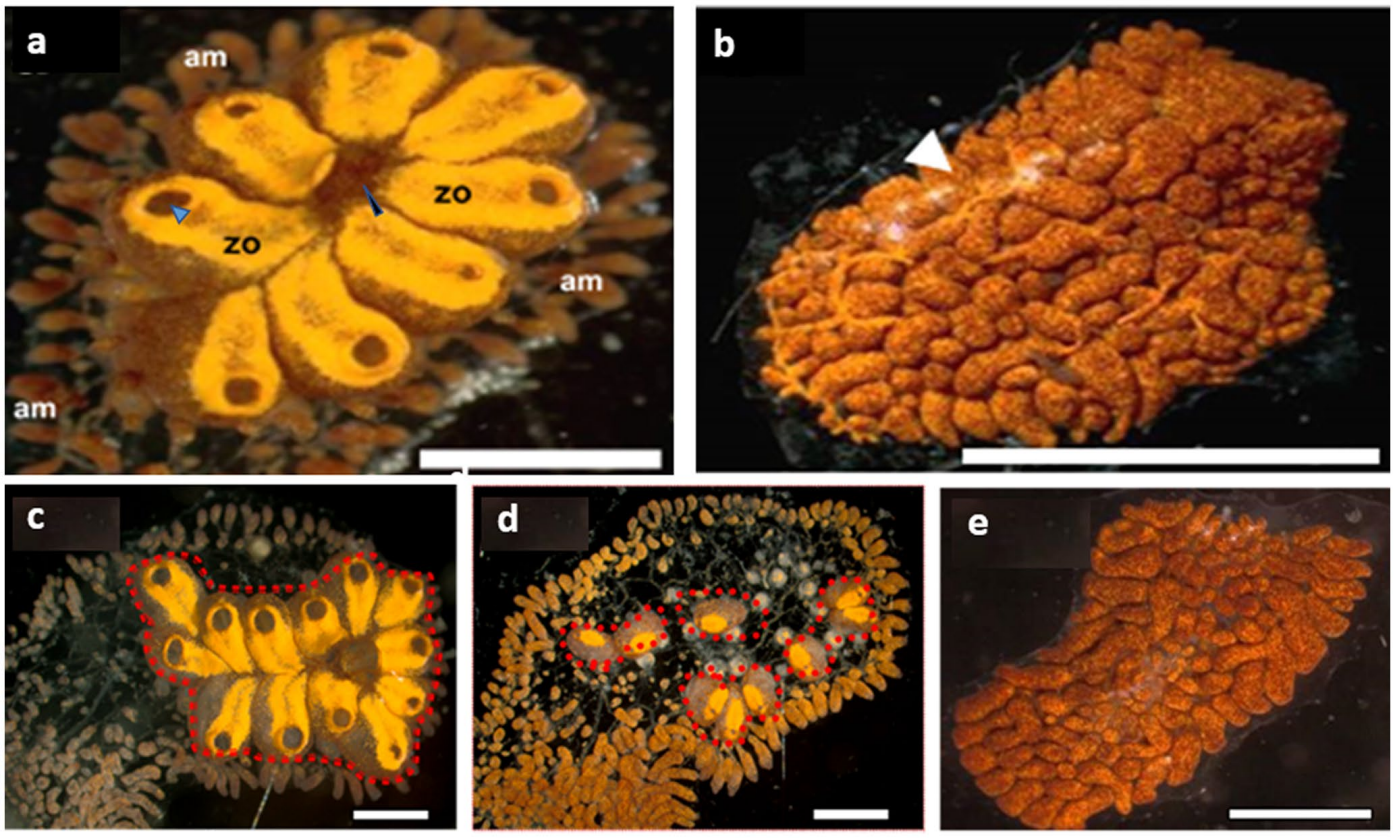
*Botrylloides leachii* torpor. **(A)** An active colony was grown on a glass slide in the laboratory. The zooids are peripherally surrounded by extended ampullae, the blind termini of vasculature that are loaded with hemocyte. Each zooid is approximately 1–1.5 mm in length and contains an oral siphon (a black arrowhead), whereas a system of 8 zooids (in this figure) shares a common atrial siphon (a blue arrowhead). The whole colony is embedded in a gelatinous matrix – the tunic. **(B)** A colony in a full hibernation state, following exposure to 15°C water temperature for 15 days (ambient seawater = 20°C). The resorbed zooids are replaced by a ‘carpet’ of opaque lacunae and dilated vasculature (a white arrowhead) loaded with pigment cells that give this colonial remnant its deep color. **(C–E)** From active **(C)**, mid torpor **(D)** to fully torpor **(E)** states in the same *B. leachii* colony. Functional units (zooids, red dash line) are located only in active and mid-torpor states. Bars = 2 mm, am = ampulla, zo = zooid.

*Botrylloides leachii* enters a state of hibernation or aestivation following an abrupt decrease or increase in ambient seawater temperature (Hyams et al., 2017, 2022). The colonies completely absorb all their functioning units (zooids), arrest bud development, and may remain for months as remnants of condensed vasculature and hemocyte lacunae (Figures 1A,B). Torpor colonies are deficient in feeding and sexual reproduction organs. When re-exposed to milder environmental conditions, tissue vestiges regenerate new fully active colonies. Arousal from hibernation starts with clear tunic areas among the vasculature lacunae, that turn into transparent buds, one of which develops into a functional zooid (Hyams et al., 2017). Hibernating colonies harbor large cells that possess rod-shaped inclusions made of electron-dense material 2–3 μm in length, which resemble bacteria (Hyams et al., 2017, 2022). These were absent in non-dormant colonies. Aiming to investigate the link between torpor, microbial diversity, and functions, we visualized microbes, sequenced DNA and RNA, curated a metagenome-assembled genome of *Endozoicomonas*, and explored the expression of its genes in cultivated *B. leachii*, during fully functional, mid- and late-torpor conditions.

## 2. Materials and methods

### 2.1. Animal husbandry

Colonies of *B. leachii* were collected from the shallow subtidal along the Israeli Mediterranean coast and were carefully peeled off the underlying surfaces of stones with industrial razor blades, to minimize tissue damage. Colonies were individually bound onto 5 × 7.5 cm glass slides using fine cotton threads and cultured at the National Institute of Oceanography, Haifa, in 25 L tanks connected to a standing seawater system (Rinkevich and Shapira, 1998). Within several days of collections, tied colonies were partially glided or relocated completely from their original calcareous substrates onto the glass slides, firmly attached to the new substrates. The colonies and the surrounding glass substrates were cleaned weekly with industrial razor blades and fine brushes. Some of these colonies were sub-cloned by cutting them into several ramets (methodology in Rinkevich and Shapira, 1998; Paz and Rinkevich, 2002) according to their size and health conditions and then re-attached to other slides, with each ramet placed onto a separate glass slide, thus allowing equal opportunity for outwards growth. Each collected colony is referred to as a distinct genotype since collected from different localities separated by at least 1 m from each other (Rinkevich and Weissman, 1987).

### 2.2. Torpor assay

Nine genotypes were divided haphazardly into three experimental groups, (a) active colonies, growing at 20°C, (b) colonies at mid torpor state following their translocation to a 15°C water tank for 5 days, and (c) colonies at full torpor status following their translocation to 15°C water tank for 20 days (Hyams et al., 2017; Figures 1C–E). The colonies were monitored daily using a stereomicroscope (Nikon SMZ1000) and photographed (Olympus XC 30).

### 2.3. Hemocytes *in vitro*

The hemocyte populations of active (*n* = 6), mid-torpor (*n* = 6) and fully torpid (*n* = 6) colonies were isolated from the vasculature (Avishai et al., 2003). Specimens were individually drained into a small petri dish, and each was stained with a fluorescent dye Hoechst 33342 (cat: 62249, Thermo Fisher Scientific) for nuclei staining. The cells were then washed several times with filter seawater (to remove excess dye) and monitored daily with a Nikon inverted phase contrast microscope for 10 days periods.

### 2.4. Histology

Control (*n* = 10), mid-torpor (*n* = 10) and hibernating (*n* = 10) colonies were fixed in Bouin’s solution for 1–2 h, dehydrated in a graded series (70–100%) of ethanol and butanol and then embedded in paraffin wax. Cross serial Sections (4–5 μm) were cut using a hand-operated microtome (Leica 2045, Nussloch, Germany), de-waxed and stained with alum hematoxylin and eosin for general morphology or with the reagent Schiff Feulgen reaction for DNA (Lillie, 1954).

### 2.5. Transmission electron microscopy

Colonial fragments containing vasculature were sampled from active (*n* = 5), mid-torpor (*n* = 3) and fully torpid (*n* = 5) colonies and were fixed in 4% EM-grade glutaraldehyde (cat: G 5882, Sigma) at room temperature, post-fixed in 1% OsO_4_ in ddH_2_O for 1 h (4°C), washed (× 3) in cold ddH_2_O and then placed in 1% Uranyl acetate in ddH_2_O overnight. Samples were then dehydrated in a graded ethanol series of 50, 70, and 95% for 10 min each, twice at 100% for 10 min each at room temperature, and then placed in Propylene Oxide (PO) for 15 min. The infiltration of Epon resin was performed by submerging samples first in 1:1 PO/Epon for 1 h, then in 1:2 PO/Epon for a night and then in 100% Epon for 2–3 h. The samples were placed in molds, labeled, filled with 100% Epon and allowed to polymerize in a 65°C oven for 24 h. Ultrathin sections (80–90 nm) were prepared with a diamond knife (Diatome) and an ultramicrotome (Ultracut, Leica), placed on 300 mesh nickel grids (Polysciences Inc.) and stained with uranyl acetate and lead citrate. The sections were analyzed using a Jeol 1230 transmission electron Microscope at 80 kV. Digital photographs were taken with a Gatan-Multiscan 701 camera.

### 2.6. DNA and RNA extractions

For DNA extraction, colonies were collected from Michmoret beach, Israel (*n* = 5 genotypes), and each was sub-cloned into three ramets. DNA was extracted from each genotype at three different time points (collected day = wild, in an active state under laboratory conditions-active, and in a torpor state under laboratory conditions-torpor), as described (Graham and Graham, 1978). RNA was extracted from 9 colonies, each at three physiological states (active, mid-torpor, and full-torpor) using an RNeasy Mini kit (QIAGEN) and digested with DNAse PurelinkTM (Invitrogen). RNA quantity was checked with a Qubit 2.0 Fluorometer and evaluated using a Bioanalyzer (Agilent). The tissue samples of active and mid-torpor states were taken from the functional units (zooids, red dashed lines Figures 1C,D) and the vasculature (the rest of the colony), and the whole fragment in a fully torpid state.

### 2.7. RNA sequencing

#### 2.7.1. Sequencing, pre-assembly checks, raw data quality and processing

DNA was sequenced from two individuals, a torpid colony and a colony in an active state. DNA library preparation and sequencing of 30 Gb 2×150 metagenomic paired-end reads using Illumina NovaSeq was performed at Novogene (Singapore). RNA (TruSeq Total RNA Library Prep Kit with Ribo-Zero Gold) Sequencing (Epicenter, Benes et al., 2011) was carried out at Helmholtz Zentrum München, Germany, using the Illumina HiSeq 2000 platform. RNA sample libraries were prepared using Illumina TruSeq™ standard total RNA Library Preparation kit. Fragments >200 bp were selected for the final enriched libraries with an average insert size of 324 ± 7 bp across all libraries. RNA Sequencing was carried out at Helmholtz Zentrum München, Germany, using Illumina HiSeq 2000 platform. Paired-end sequencing of 2 × 100 bp was carried out generating approximately 34 million reads per lane. Only 30% of the total RNA reads were annotated to *B. leachii* reference genome, whereas *circa* 15% of the reads were assigned to small subunit ribosomal ribonucleic acid (SSU rRNAs) (Blanchoud et al., 2018; Hyams et al., 2022).

### 2.8. Data analysis

DNA reads were assembled with the SPAdes V3.11 toolkit (Bankevich et al., 2012) following adapter trimming and error correction with tadpole.sh, using the BBtools suite.^1^ Downstream mapping and binning of metagenome-assembled genomes (MAGs) were performed using DAStool, Vamb, Maxbin 2.0 and Metabat2 (Wu et al., 2016; Sieber et al., 2018; Kang et al., 2019; Nissen et al., 2021). Annotation was performed using DRAM (Shaffer et al., 2020) within Atlas V2.11 (Kieser et al., 2020). SEED annotation was performed using the Rast annotation engine (Overbeek et al., 2014). An *Endozoicomonas* tree was built from 22 genomes, downloaded from PATRIC (Wattam et al., 2017), with GTOtree V1.6.37 (Lee and Ponty, 2019) using default settings (90 of 172 gammaproteobacterial single-copy targets were used) and FastTree V2.1 (Price et al., 2009), using the JTT + CAT model and SH-like 1000 bootstraps.

To assess *Endozoicomonas* expression levels at the RNA level, the transcriptome reads were mapped to the MAG with 0.97 identity cutoff using bbmap, and the expression of coding sequences was summarized using featureCounts (Liao et al., 2014). We used phyloFlash V3.4 (Gruber-Vodicka et al., 2020) to assess the phylogenetic diversity of associated microbes in transcriptomes: The total RNA libraries from three physiological states (active, mid-torpor and fully torpor) and different tissue compartments (vasculature, zooids) were mapped to the Silva 138 database (Quast et al., 2013), the mapped reads were assembled with SPAdes, and the read abundance of the full-length SSUs was calculated following remapping with BBmap using 0.97 identity cutoff. Diversity analyses were performed using the R package phyloseq (McMurdie and Holmes, 2013) and species enrichment was identified with indispecies (De Cáceres et al., 2012).

### 2.9. Bacterial marker gene sequencing

Using *Endozoicomonas* 16S rRNA gene-specific forward primer and bacteria common primer as the reverse, an 833 bp fragment specific to *Endozoicomonas* was amplified from a *Botrylloides* colony (Table 1), subcloned into pDrive vector (PCR cloning kit cat number 231124, Qiagen, Germany) and sequenced. The fragment showed 99.28 and 81.35% similarity to *Endozoicomonas* and *Fodinicurvata*. 16S rRNA gene sequences, respectively. A *Fodinicurvata* rRNA gene fragment (Table 1) was amplified using *Fodinicurvata* forward and common bacteria primers as the reverse. The subcloned *Fodinicurvata* 780 bp fragment had 99.49 and 81.5% identity to *Fodinicurvata* and *Endozoicomonas* 16S rRNA gene sequence, respectively. It also shares 92% identity to *Fodinicurvata* strain YIM D82 16S ribosomal RNA (accession number NR_044595.1).

**Table 1:** Primers used.

Organism (accession number)	Gene	Primer sequence (5′-3′)
*Endozoicomonas*	16S rRNA	Forward ACCAGGAATAGCTTGCTATTTGCTGG
		Reverse GTAGCGCAAGGTCCGAAGAGC
*Fodinicurvata*	16S rRNA	Forward ACGCGTCTTCGGACGAGTGGCGC
		Reverse GCAGCAGTTTCCCGCTGTTATTCCGTA
*Botrylloides* aff. *leachii* (MG009584.1)	18S rRNA	Forward CCATTGGAGGGCAAGTCTGGTG
		Reverse CGCGCTCGCCCAATATCCAACTAC
Bacteria common	16S rRNA	Forward ACCTTGCGGCCGTACTCCCCAGGC

The subcloned *B. leachii* 18S fragment (Table 1) was 109 bp and had 100% identity to the 18S gene of *Botrylloides* aff. *leachii* (accession number MG009584.1).

### 2.10. qPCR

Relative qPCR analyses were performed on standards and DNAs extracted from tested tissues with Fast SYBR™ Green Master Mix (cat.no 4385614; Thermo Fisher Scientific, MA, United States) using StepOnePlus Real-Time PCR System (Thermo Fisher Scientific, MA, United States). Specific standard curves for *Endozoicomonas* and *Fodinicurvata* 16S and *B. leachii* 18S rRNA genes were established by amplifying 4 known amounts of each of the standard DNAs (concentrations: 0.1, 0.01, 0.001, 0.0001 ng/mL). On the same plate, the quantities of *Endozoicomonas* and *Fodinicurvata* 16S and *B. leachii* 18S rRNA genes were analyzed in ramets originating from colonies at three different states: wild (*n* = 5, collected from Michmoret beach), active (*n* = 5, acclimatized in the institutional mesocosm) and hibernating colonies *n* = 5. Using the standard curves (concentration vs. CT; 69), the quantities of specific 16S genes (proportional to bacteria quantities) were extrapolated for each tissue and normalized to rRNA gene quantity (proportional to *B. leachii* tissue quantity). Statistical analysis was performed using a one-way ANOVA between the different groups.

## 3. Results and discussion

### 3.1. Intracellular bacteria are enriched in torpid *Botrylloides aff. leachii*

**Figure 2:**
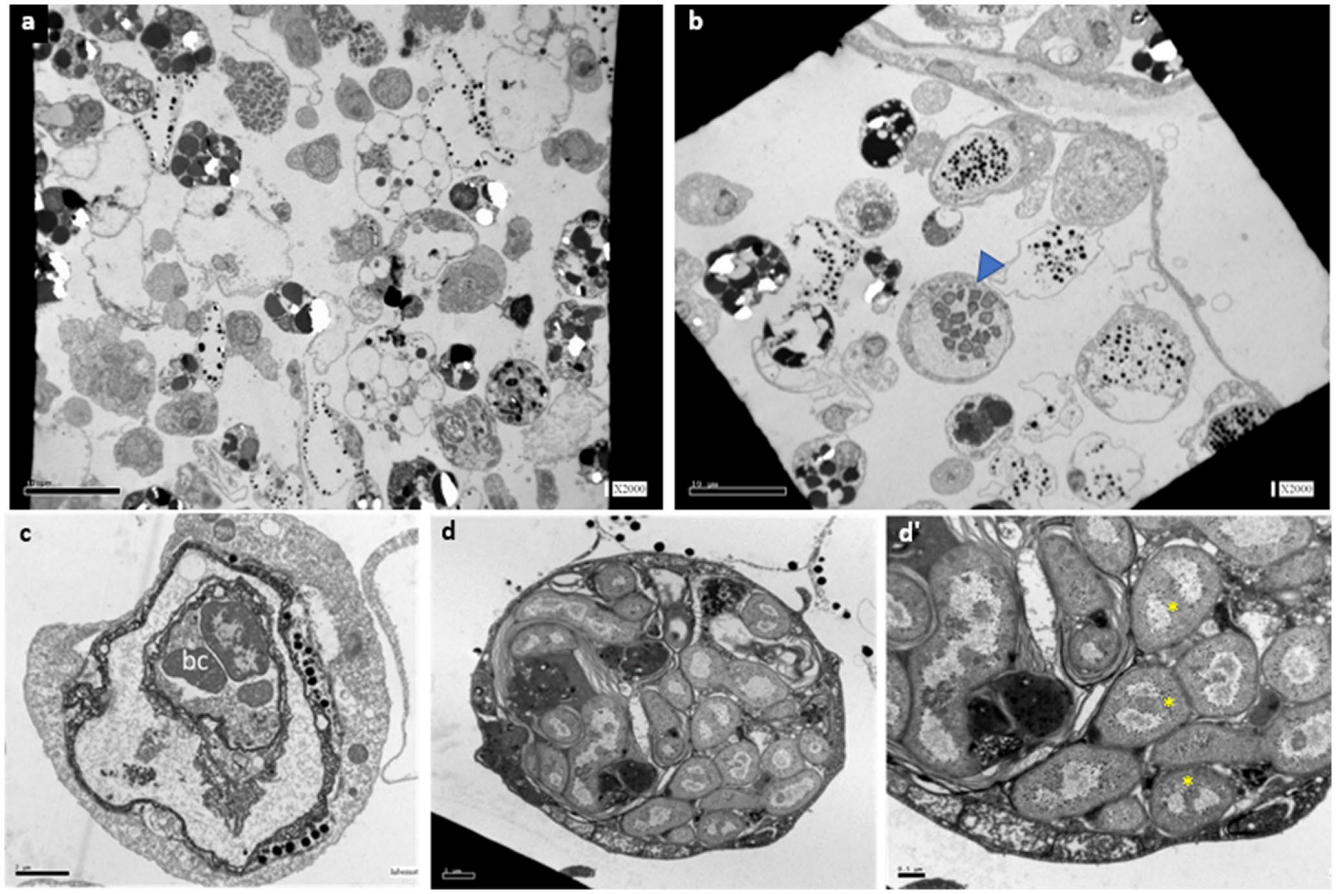
TEM sections of *B*. aff. *leachii* vasculature and hemocytes in active, mid-torpor and fully torpid states. **(A)** An active phenotype, no cell-containing bacteria. Bar-10 μm. **(B)** A torpid colony, bacteria cells within the cytoplasm of some circulated hemocytes (a blue arrowhead). Bar-10 μm. **(C)** Bacteria-containing cells, phenotype 1-a 12 μm animal cell containing 2 vacuoles, the inner vacuole holds 3 intact bacteria cells. Bar-2 μm. **(D)** A bacteria-containing cell, phenotype 2- a 22 μm cell holding 22 intact bacteria cells. **(D’)** The bacteria cell wall is double layered, as some of the naked DNA that is at the state of binary fission (yellow asterisks). Bar-0.5 μm.

Transmission electron microscopy (TEM) observations in active (*n* = 5), mid-torpor (*n* = 3), and fully torpid (*n* = 5) colonies revealed bacteria associated with cells in the vasculature of only mid and full-torpid states (Figure 2, 2–8% of the total circulating cells in mid- and fully torpid states, respectively), as indicated previously (Hyams et al., 2017, 2022). The size of the host cells containing intracellular bacteria was 8–30 μm. We found two distinct cell phenotypes with bacteria: (i) Cell sizes 10–20 μm, where most of the cell volumes are occupied by a few, 1–3 vacuoles (8–14 μm in length), and smaller vacuoles inserted within larger vacuoles. Each vacuole contained 3–15 bacteria cells of 1–2 μm, each (Figure 2C). With nuclei and mitochondria located at the periphery. The bacterial cells within the vacuoles were intact (Figure 2C). (ii) Cell sizes of 10–30 μm. We observed multilayer vacuoles that accounted for a large fraction of the cell volume. Each host cell contained 15–20 intact, 1–2 μm long, bacterial cells (Figure 2D). All the bacteria within the host cells were morphologically intact and well organized within the cells, some of them were found in a binary fission state (Figure 2D’, yellow asterisks). As in phenotype 1, the nuclei of host cells and mitochondria in phenotype 2 were in the cell periphery.

**Figure 3:**
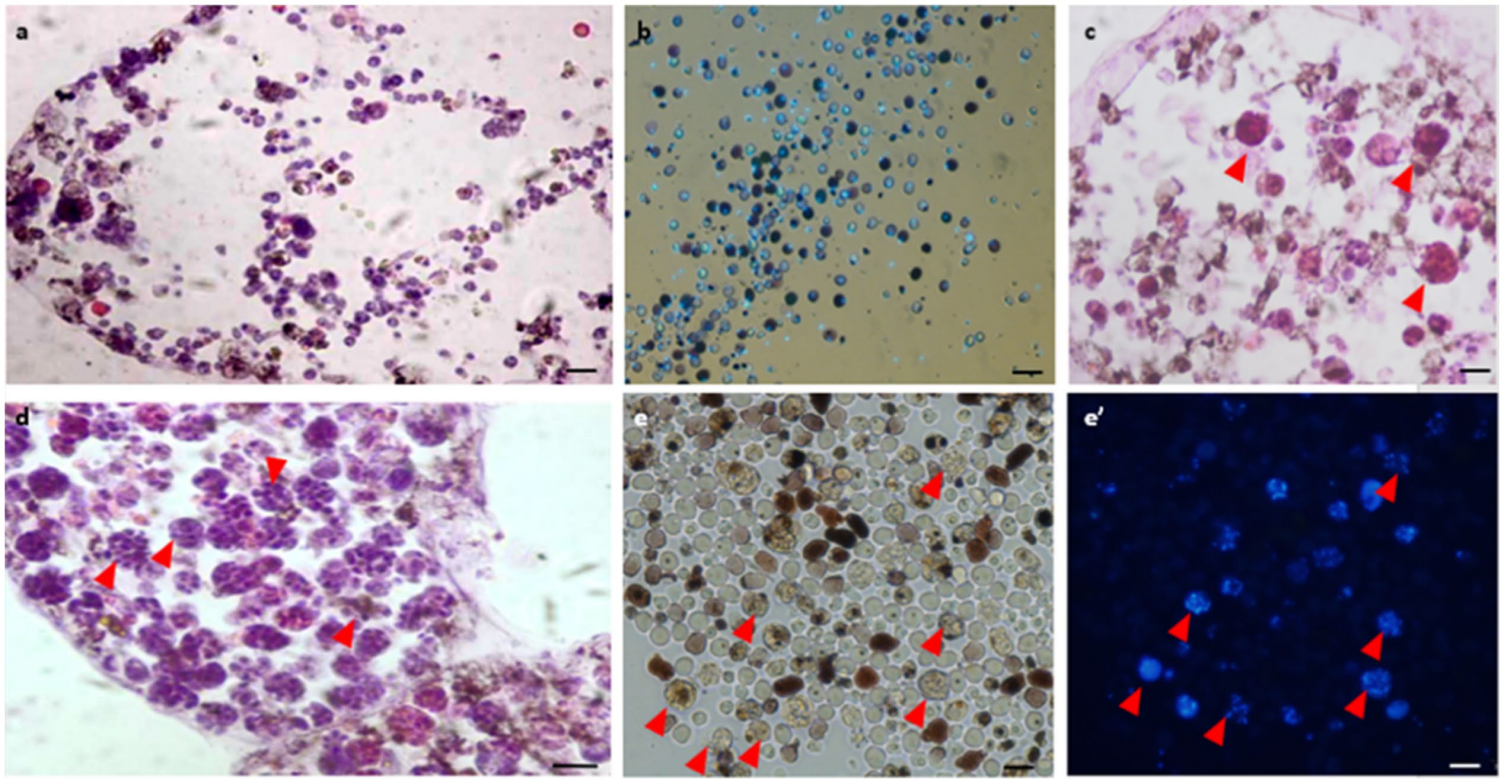
Histological observations of vasculature and hemocytes in active, mid-torpor and fully torpid colonies. **(A)** Active colony- histological section through a peripheral ampulla stained with hematoxylin & eosin. **(B)** *In vitro* Hoechst stain of hemocytes isolated from the active colony. **(C)** Mid-torpor colony- histological section through a peripheral ampulla stained with hematoxylin & eosin. Several hemocytes containing multiple DNA spots are marked (red arrowheads). **(D)** Torpid colony- Histological section through an ampulla (hematoxylin & eosin). A wide range of hemocytes with multiple DNA spots (red arrowheads). **(E)** *In vitro* Hoechst-stained hemocytes isolated from a torpid colony **(E’)**, many of them contain multiple DNA spots (marked with red arrowheads). Bars- 10 μm.

Histological observations performed on active colonies (*n* = 12) and in Hoechst-stained isolated cells showed no evidence of bacteria within either vasculature or in isolated hemocytes under *in vitro* conditions (Figures 3A,B). In mid-torpor colonies (*n* = 6), 1–4% of the hemocyte populations appeared as large hemocytes (10–15 μm) containing several stained ‘nuclei’ (2–13) within each cell membrane, suggesting the appearance of bacteria cells (Figure 3C, red arrowheads). In the fully torpid colonies (*n* = 8), high proportions (10–70%) of the hemocyte populations appeared as large hemocytes (10–30 μm) populated by ‘nuclei’ (*n* = 5–15), further suggesting that some of the multinucleate cells are of bacteria inclusive cells (Figures 3D,E,E’, red arrowheads). Only TEM observations were able to differentiate between multinucleate cells and cells with bacteria.

### 3.2. Microbiota composition changes in active, mid torpor and fully torpid *Botrylloides leachii* colonies

To study changes to the microbiome during torpor, we analyszed our previous RNA-sequencing data (Hyams et al., 2022), this time for bacterial 16S rRNA gene sequences.

**Figure 4:**
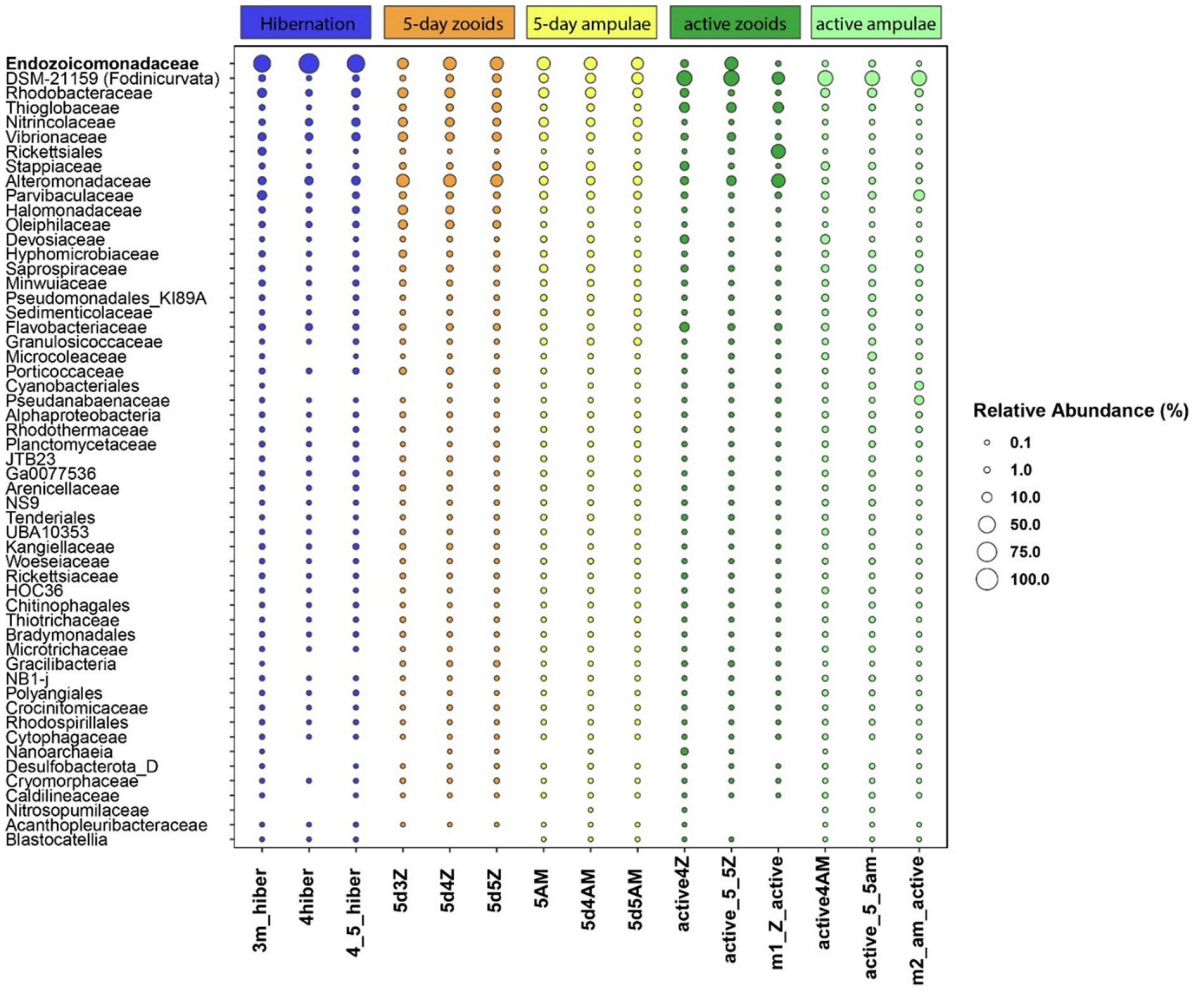
The relative read abundance of 54 bacterial taxa in the vasculature of torpid colonies, mid-torpor state (5 days) and active colonies (3 different biological replicates for either physiological state, each replicate marked by its ID at the *x*-axis base). Empty slots: relative abundance <0.1%. Taxonomy is based on the annotation of the full, SPAdes-assembled rRNA sequences, classified using the best BLAST hit in the Silva 138 database.

**Figure 5:**
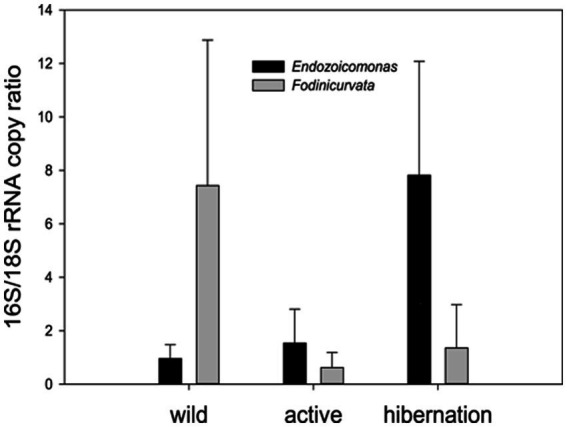
qPCR quantification of the two key bacteria associated with *B. leachii* hibernation– the ratio between the copy numbers of the rRNAs: bacterial 16S to the host 18S. *Endozoicomonas* was significantly enriched in the hibernation state (*n* = 5), compared to freshly collected (wild *n* = 5) and laboratory-grown samples (active *n* = 5). *Fodinicurvata* was highly abundant in some freshly collected samples. One-way ANOVA: *Endozoicomonas* along different stages *F* (2,12) = 9.915, *p* = 0.0038 *Fodinicurvata F*(2,12) = 1.913, *p* = 0.19.

**Figure 6:**
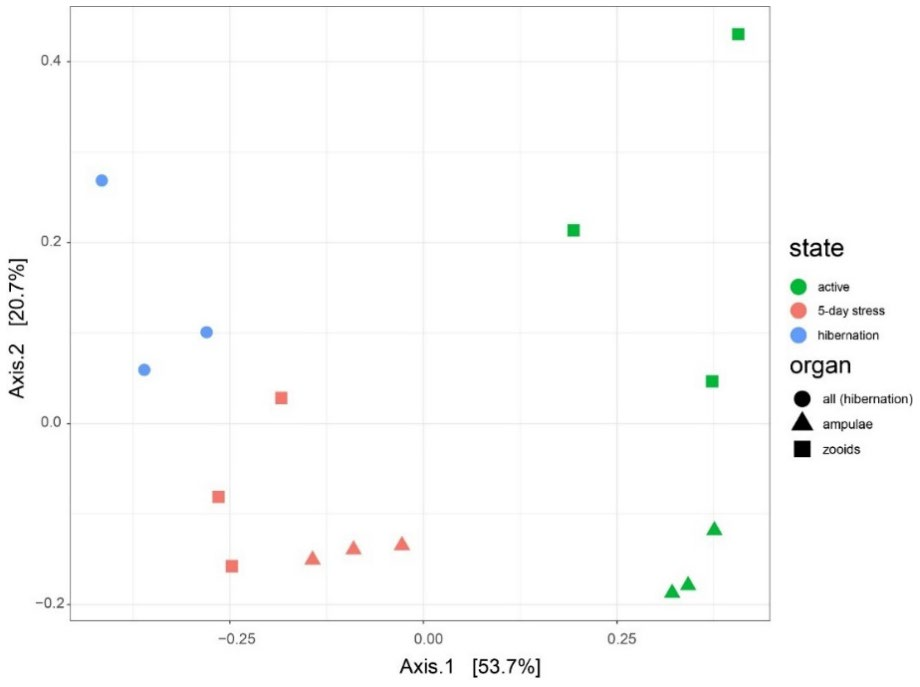
Microbiota varies between different physiological states (active, mid-torpor, and full-torpor colonies) and tissue types (vasculature and zooids) in *Botrylloides leachi.* Principal Coordinates Analysis (PCoA) based on the Bray–Curtis dissimilarity matrix of transcriptomic rRNA full-length read abundances.

We found 54 full-length 16S rRNA sequences of bacteria and archaea associated with *B. leachii*, based on transcriptomics (Figure 4), Taking into consideration that laboratory conditions may also affect the microbiome. The key lineages included Endozoicomonadaceae that were not identified beyond the family level, alphaproteobacterial taxa such as Fodinicurvata sp., as well as Thioglobaceae (SUP05), Nitrincolaceae, Rikketisiales and Vibrionaceae lineages, some of which tend to be found in symbiotic associations (Merhej and Raoult, 2011; Neave et al., 2016; Perez et al., 2022), including those of ascidians (Schreiber et al., 2016). Enrichment analyses revealed that in full torpor the read abundance of *Endozoicomonas* was the highest (53–79% of all bacterial reads were annotated as Endozoicomonas compared to 13–25% in mid torpor zooids and just 0.14–1.76% in active colonies, indicator species analysis statistic [indicpecies stat.] value 0.90, *p* < 0.001). In the active state, Fodinicurvata read abundance was 21–39% in zooids and 34–38% in the vasculature, as compared to <2% of all reads in the vasculature of fully torpid colonies (indicpecies stat. value 0.94, *p* = 0.001). We observed enrichment of Thioglobaceae sp. in zooidal tissues, primarily in regular colonies (9.58–11.93% in active zooids, as compared to 1.29–1.72% in the vasculature). Thioglobaceae abundance was reduced in zooids of mid-torpid zooids to 2.02–7.41% (1.51–2.92% in the vasculature). In fully torpid fragments Thioglobaceae read abundance was only 0.1–0.65% (Supplementary Table 1). We verified the Endozoicomonas enrichment during torpor using DNA-based PCR. The ratio of Endozoicomonas 16S and Botrylloides rRNAs was significantly higher in hibernating colonies (*p* < 0.001) than in individuals collected directly from the sea and active colonies from the laboratory (Figure 5). In the specific qPCR performed, these ratios were not significantly different (*p* > 0.1) between the different physiological states for Fodinicurvata. These results indicated that the *B. leachii* microbiota does not remain stable during torpor progression, and specific lineages prevail in torpor (explaining 74.4% of the variation; Figure 6). Here after we focus on the Endomonazaicoceae symbiont, describing its phylogeny and function, because of its enrichment in the torpor state.

### 3.3. The functionality of the symbiotic *Endozoicomonas* lineage in torpor and mid-torpor *Botrylloides leachii*

*Endozoicomonas* reads were found only in the torpor library, and 238,000 reads were mapped to Metagenome-Assembled Genome (13x coverage). The total length of the genome was 2.64 Mb, comprising 165 contigs with an N50 value of 23610. Checkm (for assessing the quality of genomes) indicated that the genome is 90% complete with 0.8% contamination, whereas Checkm2 suggested 100% completeness and 0.1% contamination.

**Figure 7:**
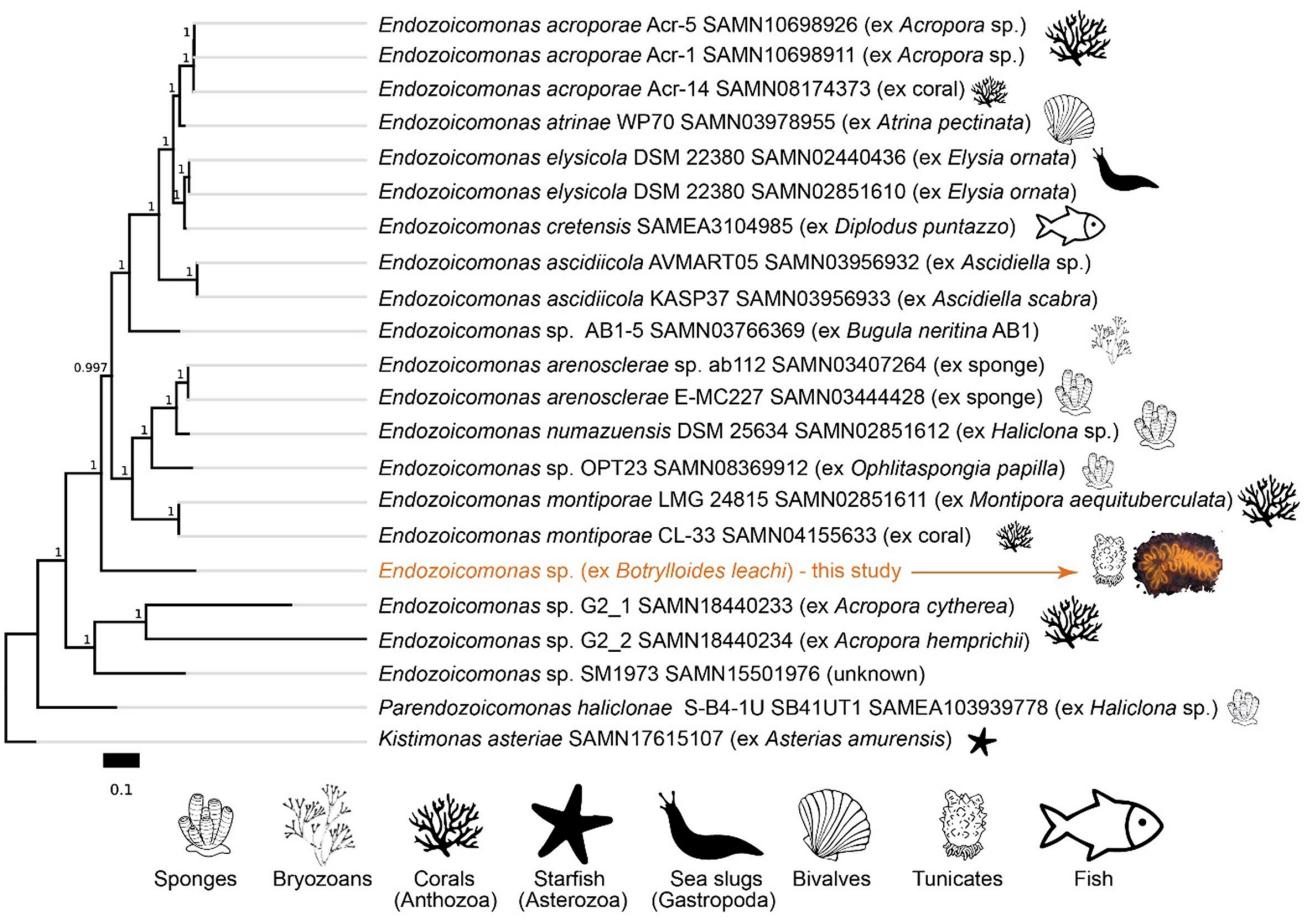
Phylogeny of *Endozoicomonas* species based on amino acid sequences of 90 single-copy gene markers (maximum likelihood, JTT + CAT model). The scale bar represents the number of substitutions per site. Branch bootstrap support values are shown. NCBI BioSample accession numbers are indicated for the respective genomes.

Metagenomics, and analysis of the 16S rRNA sequence, suggest that *B. leachii* hosted a distinct lineage of *Endozoicomonas*. Its 16S rRNA sequence had 94.87% identity with the best hit in the NCBI’s database, the sequence from the *Endozoicomonas montiporae* CL-33 genome (CP013251.1). *Endozoicomonas* sp. ex *B. leachii* MAG is basal to a large branch of Endozoicomonadanceae genomes that are associated with distinct hosts (Figure 7). Phylogenomic treeing suggested multiple host switches within the *Endozoicomonas* clade. In particular, the ascidian-associated *Endozoicomonas ascidiicola* (Schreiber et al., 2016) is not the closest relative of *Endozoicomonas* sp. ex *B. leachii*. Given the highest average nucleotide identity of 70% between the MAG of *Endozoicomonas* sp. ex *B. leachii* and other genomes in our comparison, we suggest that this lineage represents a novel candidate species, which we propose to name *Candidatus* Endozoicomonas endoleachii.

**Figure 8:**
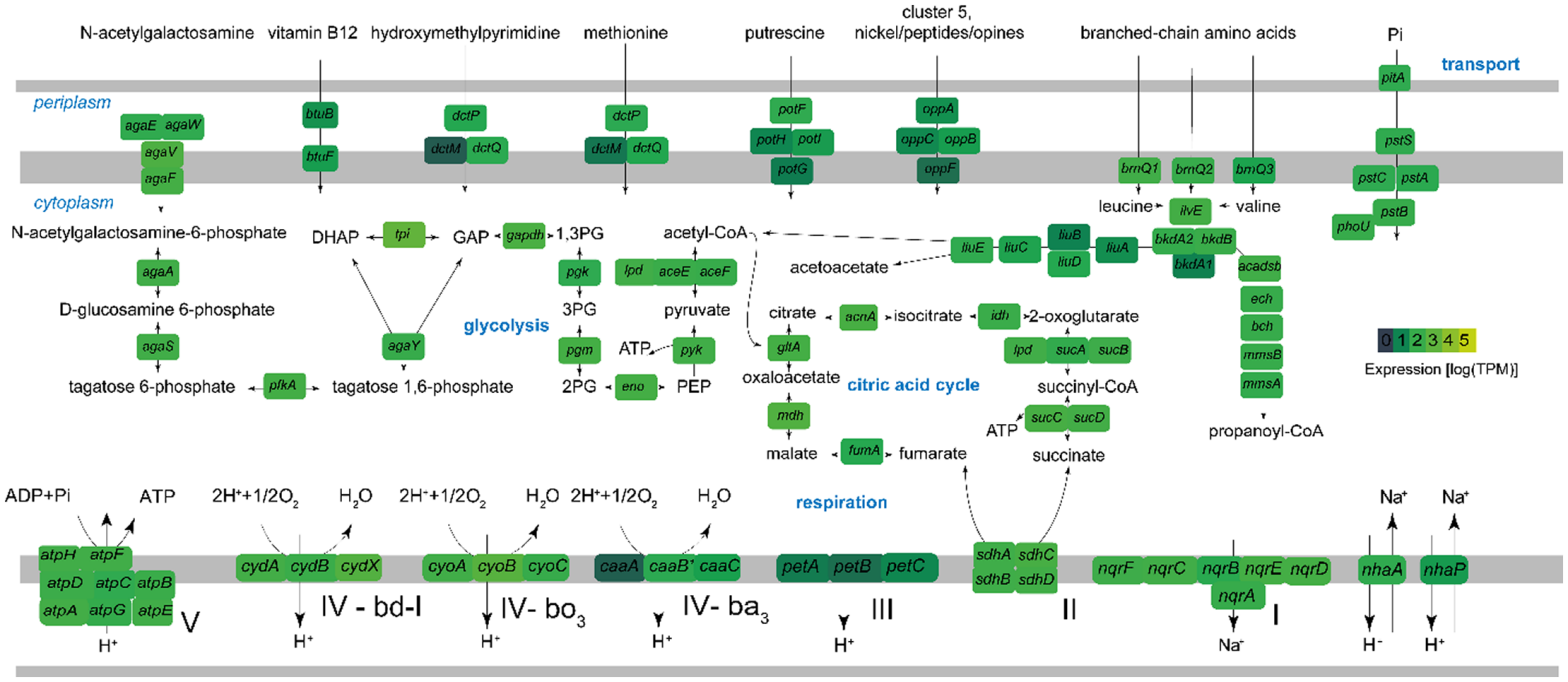
Key metabolic pathways of *Ca.* Endozoicomonas endoleachii, based on genome-centered transcriptomics. The enzymes that catalyze each reaction are marked with the name of the encoding gene. Colors represent gene expression only in torpor colonies, as log transcripts per million reads (TPM). The following enzymes and respective genes are shown: PTS system, N-acetylgalactosamine-specific IIC component *agaW*; PTS system, N-acetylgalactosamine-specific IID component *agaE*; PTS N-acetylgalactosamine transporter subunit IIB *agaV*; N-acetylgalactosamine transporter subunit IIA *agaF*; N-acetylgalactosamine-6-phosphate deacetylase *agaA*; D-galactosamine-6-phosphate deaminase *AgaS*; 6-phosphofructokinase *pfkA*; tagatose-bisphosphate aldolase *agaY*; triosephosphate isomerase *tpi*; glyceraldehyde-3-phosphate dehydrogenase *gapdH*; phosphoglycerate kinase *pgk*; phosphoglucomutase *pgm*; enolase *eno*; pyruvate kinase *pyk*; pyruvate dehydrogenase *aceEF-lpd*; citrate synthase *gltA*; aconitase *acnA*; isocitrate dehydrogenase *idh*; 2-oxoglutarate dehydrogenase *sucAB-lpd*; succinyl-CoA ligase *sucCD*; succinate dehydrogenase *sdhABCD*; fumarate hydratase *fumA*; malate dehydrogenase *mdh*; F_0_F_1_-type ATP synthase *atpABCDEFGH*; Na(+)-translocating NADH-quinone reductase *nqrSBCDEF*; Na(+)/H(+) antiporters *nhaA* and *nhaP*; cytochrome bc1 complex *petABC*; cytochrome bd-I ubiquinol oxidase *cydABX*; cytochrome bo_3_ ubiquinol oxidase *cyoABC*; ba_3_-type cytochrome c oxidase *caaABC*; nranched-chain amino acid permeases *brnQ*; branched-chain-amino-acid aminotransferase *ilvE*; branched-chain alpha-keto acid dehydrogenase complex *bkdA1A2B*; isovaleryl-CoA dehydrogenase *liuA*; methylcrotonyl-CoA carboxylase *liuBD*; methylglutaconyl-CoA hydratase *liuC*; hydroxymethylglutaryl-CoA lyase *liuE*; acyl-CoA dehydrogenase short/branched chain *acadsb*; enoyl-CoA hydratase *ech*; 3-hydroxyisobutyryl-CoA hydrolase *bch*; 3-hydroxyisobutyrate dehydrogenase *mmsB*; methylmalonate-semialdehyde dehydrogenase *mmsA*; phosphate transport system *pstABC-phoU-pltA*; oligopeptide ABC transporter *oppABCF*; putrescine transporter *potFGHI*; ABC transporters *dctMPQ*; vitamin B12 transporter *btuBF*.

The metagenome-assembled genome investigation suggested that *Candidatus* E. endoleachii exhibits a heterotrophic lifestyle, probably focused on the turnover of proteins, amino acids and their derivatives (18 and 19% predicted SEED functions, respectively, Supplementary Figure 2), whereas 11% of the genomic features are dedicated to cofactors, vitamins, prosthetic groups and pigments, and 7% to carbohydrates. The tricarboxylic acid cycle appeared to be complete and highly expressed (the *mdh* gene encoding malate dehydrogenase was among the top 25 most expressed genes; Figure 8 and Supplementary Table 1). Glycolysis enzymes were moderately to highly expressed at the mRNA level (Figure 8). *Endozoicomonas* genome (Deutscher et al., 2006) carries more than 10 genes, most of which cluster and are highly expressed, encoding a phosphotransferase system (PTS), which allows transport of PTS-sugars, often preferred bacterial substrates (Deutscher et al., 2006). This system is likely specific to N-acetylgalactosamine (GlcNAc), as *Ca.* E. endoleachii encodes and expresses the *aga* operon (Brinkkötter et al., 2000), further allowing the symbiont to take advantage of glycans that appear to be enriched in tunicates (Zeng et al., 2022). Gene clusters encoding the glycine cleavage system, methionine transport, arginine/ornithine transport and degradation and branched-chain amino acid degradation, as well as the *oppABCF* genes (ABC oligopeptide transporter), were found to be expressed substantially (Figure 8 and Supplementary Table 1). Thus, *Ca.* E. endoleachii appears to be capable of glycogen storage. These features may allow *Endozoicomonas* the commensal or parasitic lifestyle within the *B. leachii* colonies. Three types of terminal oxidases were found (ba_3_, bo_3_, and bd-I), hinting at adaptation to changes in oxygen availability. We could not identify genes involved in the use of testosterone or dimethylsulfoniopropionate, as has been suggested for *E. montiporae* (Ding et al., 2016) and *E. acroporae* (Tandon et al., 2020). However, the genome is only 90% complete based on the CheckM estimate (Parks et al., 2015) and has a smaller size than most *Endozoicomonas* (2.7 as opposed to ~4–7 Mb), thus some genomic features may be missing.

*Ca.* E. endoleachii has several genomic features that could facilitate biotic interactions. These include genes that encode multiple features of type II, III, and VI secretion systems. Similar to Endozoicomonadaceae found in other hosts (Tandon et al., 2020), *Ca.* E. endoleachii encoded and expressed two eukaryotic repeat proteins, 14117 and 15557 nucleotides in length (data not shown). Conserved domain search revealed that these proteins comprised multiple cadherin-like, Ig-like, VCBS domains, as well as tandem-95 repeats, a carbohydrate-binding module NPCBM/NEW2 and a type I secretion C-terminal target domain.

We found gene clusters needed for the synthesis of biotin and thiamine diphosphate vitamins, the latter of which depend on the transport of hydroxymethylpyrimidine based on the presence and substantial expression of *thiX-thiY-thiZ* genes clustered with the *thiD* gene which encodes the hydroxymethylpyrimidine kinase (Supplementary Table 1; Rodionov et al., 2002). Thus, the interaction between the *Endozoicomonas* symbiont and the host may not be fully commensal or parasitic, as some metabolic trade-offs may be beneficial to *B. leachii* host.

**Figure 9:**
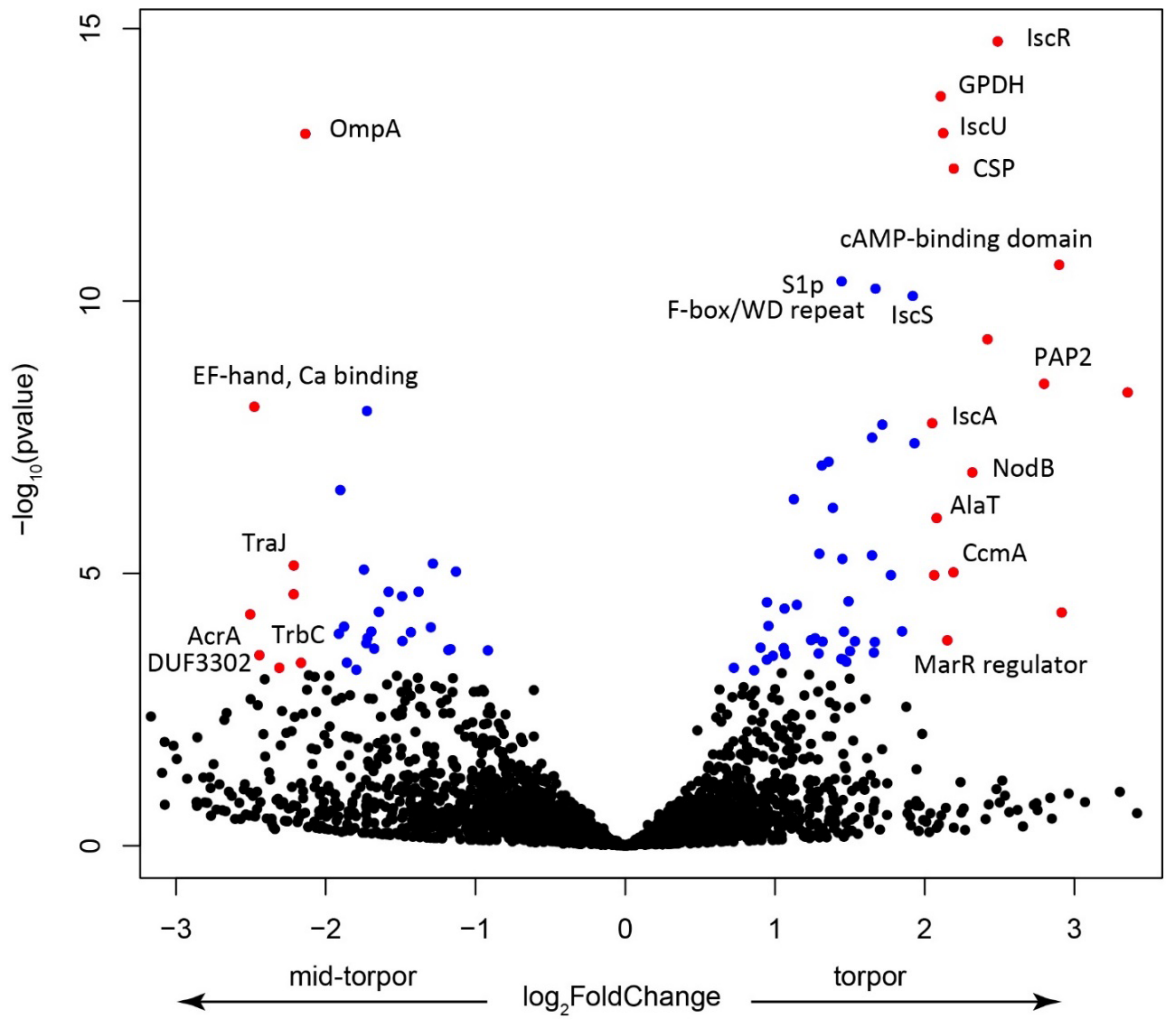
Volcano plot showing the key features upregulated in the mid-torpor and torpid states. Each dot represents a gene, blue if the adjusted *p* value < 0.01, red if log2 fold change >1 and adjusted *p* value < 0.05. Selected genes were annotated using the protein product nomenclature. GPDH is glycerol-3-phosphate dehydrogenase [NAD(P)^+^] (EC 1.1.1.94). CSP is a cold shock protein. PAP2 is a putative membrane-associated phospholipid phosphatase, PAP2 superfamily.

We investigated which *Endozoicomonas* genes were overexpressed during torpor. Because in active, non-torpor, individuals, very few RNA reads mapped to *Endozoicomonas*, we focused on comparing expression in libraries derived from mid-torpor and full-torpor individuals. DESeq2 analysis revealed marked differences in expression profiles between these two stages (Supplementary Figure 1). The key genes that were upregulated in full torpor belonged to the *isc* operon (Figure 9), which is required for the synthesis of Fe-S cluster proteins and plays a major role in pathogenicity (Lim and Choi, 2014). The key sensory gene in this operon, *IscR*, was that with the strongest upregulation (Figure 9). IscR is essential for the virulence of the *V. vulnificus* pathogen, and its exposure to host cells induces the expression of this sensory gene (Lim and Choi, 2014). Thus, the synthesis of Fe-S cluster proteins likely plays an important, yet unknown role in the interaction between *Endozoicomonas* and its host. It is not surprising that one of the most upregulated *Endozoicomonas* genes in full-torpor hosts is the one that encodes a cold shock protein, potentially responding to the ambient temperature of 15°C.

In the mid-torpor state, the most overexpressed gene encoded the putative outer membrane protein OmpA (Figure 9), a small β-barrel membrane anchor that establishes a physical linkage between the outer membrane and peptidoglycan layer (Buchanan, 1999). OmpA may play a pivotal role in bacterial pathogenesis (Mishra et al., 2020). These Omp proteins are often highly expressed in symbiotic systems, such as that of the tubeworm *Riftia pachyptila* and its gammaproteobacterial endosymbiont, where they may be involved in biotic interactions (Hinzke et al., 2021). Given that this gene is overexpressed during torpor onset when *Endozoicomonas* could establish its population, it may also be involved in the interaction with a more active host, in particular with its immune system. We also observed the upregulation of several genes that encode elements of conjugative transfer (Glöckner et al., 2008), which indicates potential activation of the mobilome and the possibility of an increased gene transfer, which increases pathogenicity.

### 3.4. The potential role of *Botrylloides leachii* endosymbionts in active and torpor hosts

*Botrylloides leachii* hosts a diverse microbial community, some of which may have a tight symbiotic partnership with the host. The dominant bacterial species in active colonies, in the and zooids, is *Fodinicurvata sediminis*, a gram-negative, facultatively anaerobic and non-motile bacterium. These bacteria secrete enzymes like urease and arginine dihydrolase, and their substrates are most probably provided by the host’s metabolic processes. While the growth temperature range for *Fodinicurvata* is 15–42°C (optimum, 28°C; 81), the winter simulating low 15°C seawater temperature that is associated with the torpor state, might be the explanation for their dramatic decrease. Unfortunately, we could not assemble a high-quality genome of *Fodinicurvata*, and the exploration of its function, as well as that of multiple low-abundance microbes, is pending.

The transition from the active to torpor states is characterized by a dramatic morphological, physiological and cellular changes within a few days, from an active colony with multiple functional zooids to small remnants of vasculature characterized by sluggish hemocyte circulation and no functional zooids (Hyams et al., 2017, 2022 and the present results). Under these non-feeding conditions, the torpor state may last for several months (Hyams et al., 2017). In the hibernating state, the bacteria appear to concentrate in an intimate special environment (within cells; Figure 2), that may be required for the holobiont functioning during the torpor. Upon arousal from torpor, no bacterial inclusions are found (Hyams et al., 2017), and their numbers are reduced dramatically. The symbiotic bacteria in this niche may either play beneficial roles, such as aiding nutrition, or use this unusual physiological state to its advantage, like outcompeting other bacteria, or deal with the animal’s immune system.

We observed considerable changes in microbial composition during the transition from the active state to the full-torpor state. *Endozoicomonas* appeared to be the most abundant symbionts in the torpor state. In various hosts, these symbionts may facilitate antimicrobial activity and microbiome structuring (Gardères et al., 2005; Morrow et al., 2012; Jessen et al., 2013; Rua et al., 2014), and be involved in metabolite exchange (Raina et al., 2009; Bourne et al., 2013; Correa et al., 2013; Nishijima et al., 2013; Beinart et al., 2014; Dishaw et al., 2014; Haber and Ilan, 2014). In particular, *Endozoicomnas* from the solitary tunicate *Ciona intestinalis* is likely involved in sulfur cycling and nutrient metabolism (Dishaw et al., 2014). It is still unclear if *B. leachii* host benefits from maintaining *Endozoicomonas* during torpor. One advantage could be the supply of vitamins, needed to maintain the hibernating host. While less likely, given that our results suggest enhanced mortality during the torpor state (Hyams et al., 2017, 2022), it may also be postulated that *Endozoicomonas* benefits from the reduction in the host resilience, and proliferates, out-competing other bacteria that may become limited by food influx in an inactive animal. Then, the intracellular *Endozoicomonas* (Figures 2, 3) are not limited by food influx as focusing on the scavenging of metabolites within the cells.

## 4. Conclusion

This study shows for the first time that facultative, potentially intracellular symbionts such as *Endozoicomonas* may prevail during a torpor state in a urochordate species. We expanded the current knowledge of phylogenetic and functional diversity in the symbiotic *Endozoicomonas* clade, showing that autocatalytic and nutritional-associated features are present and may be upregulated during the torpor state. *Endozoicomonas* likely plays an important role in the fitness of the holobiont, yet it is unclear if this role is beneficial. Given that these symbionts appear to occupy only a certain tissue type during torpor, our study raises new questions regarding the role of the vascular tissue that composes the entire soma during torpor states. As torpor is frequently recorded in various marine invertebrates and vertebrates (Crawshaw et al., 1985; Cáceres, 1997; Miller and Roth, 2009; Storey and Storey, 2011; Chen et al., 2016), it is highly plausible to find similar torpor-associated symbioses in other marine invertebrates. Thus, more studies should focus on similar torpor scenarios in the marine arena, aiming to shed light on this abrupt response to hostile environmental conditions.

## Data availability statement

The datasets presented in this study can be found in online repositories. The names of the repository/repositories and accession number(s) can be found in the article/Supplementary material.

## Author contributions

YH and BR conceived and designed the experiments. YH and AR performed the experiments. BR, YR, and MR-B provided resources. YH and MR-B analyzed the data. YH, BR, and MR-B wrote the manuscript with the contributions of all co-authors. All authors contributed to the article and approved the submitted version.

## Conflict of interest

The authors declare that the research was conducted in the absence of any commercial or financial relationships that could be construed as a potential conflict of interest.

## Publisher’s note

All claims expressed in this article are solely those of the authors and do not necessarily represent those of their affiliated organizations, or those of the publisher, the editors and the reviewers. Any product that may be evaluated in this article, or claim that may be made by its manufacturer, is not guaranteed or endorsed by the publisher.

## Funding

This study was funded by the United States–Israel Binational Science Foundation (BSF No. 2015012; to BR), by the e BSF, as part of the joint program with the NSF, the National Science Foundation, USA (NSF/BSF no 2021650; to BR), by the ISF grant No. 172/17 to BR and by the ISF grant 913/19 to MR-B.

## Footnotes

^1^sourceforge.net/projects/bbmap/
