# Supplementary material for: Physiological changes during torpor favor association with Endozoicomonas endosymbionts in the urochordate Botrylloides leachii
Source: Front Microbiol. 2023 May 31;14:1072053. doi: 10.3389/fmicb.2023.1072053 (PMC10264598; doi:10.3389/fmicb.2023.1072053)

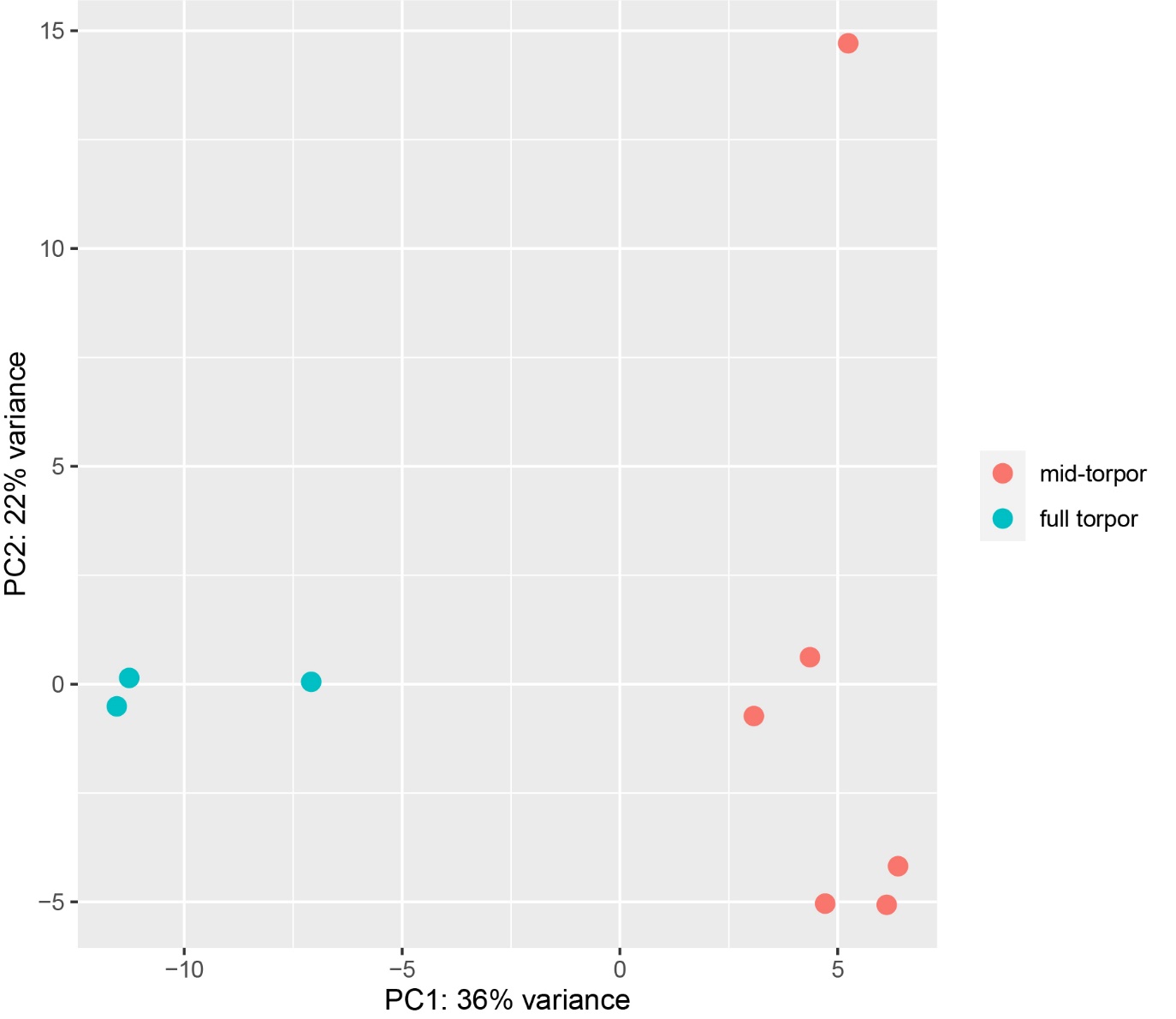


**Supplementary Fig. 1**: Principal component analysis of normalized expression profiles in *Endoicomonas* symbionts of *Botrylloides leachi*.

**Supplementary Fig. 2**- SEED features distribution in Pie chart of Endozoicomonas along torpor stage. Total of 947 reads count that are divided into 27 categories (in parentheses- numbers of reads/category)


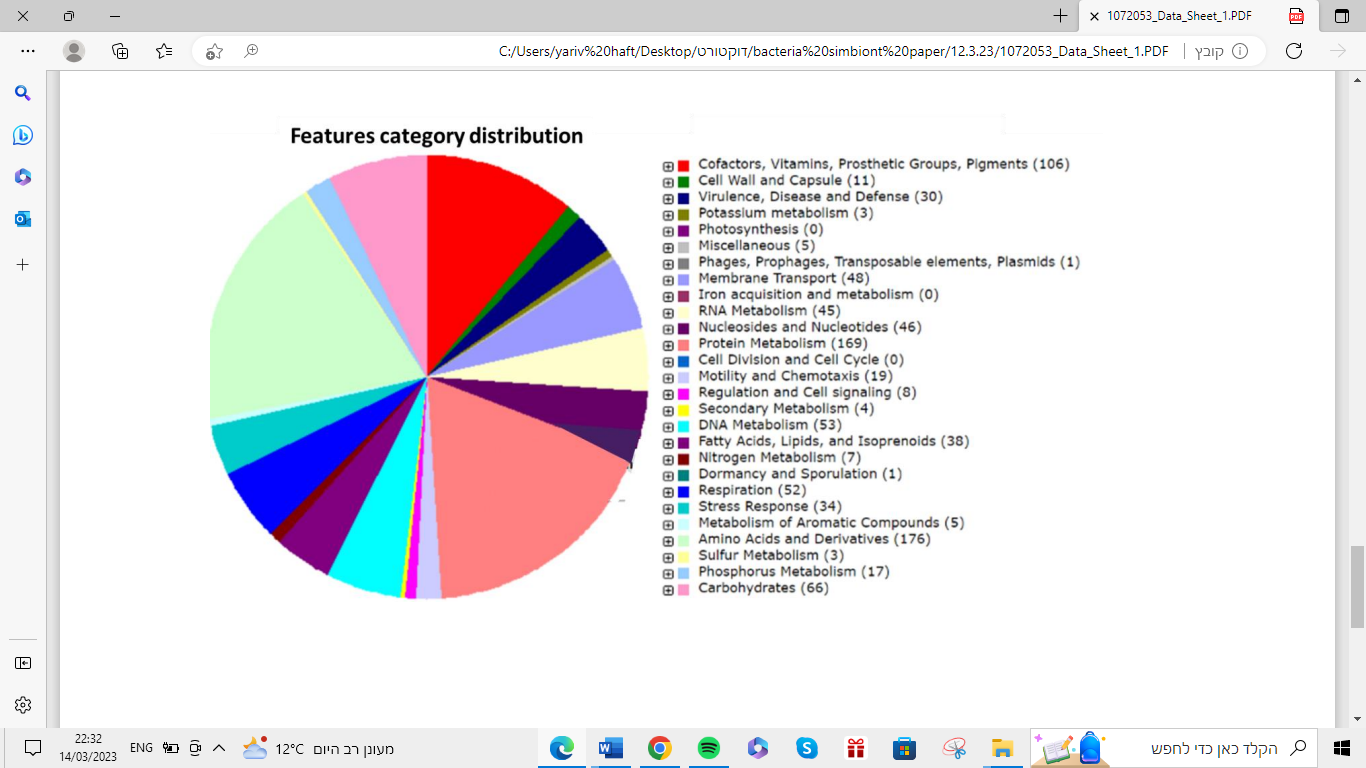

Supplement: Supplementary file 2 [file Data_Sheet_1.docx]
